# Development of a Novel Statistical Model for Predicting Clinical Outcomes in Stroke Patients With Tandem Occlusions After Endovascular Therapy

**DOI:** 10.7759/cureus.59703

**Published:** 2024-05-05

**Authors:** Sanjeev Nayak, Lucy Grant, Vias Demetriou, Marko Raseta

**Affiliations:** 1 Neuroradiology, Royal Stoke University Hospital, Stoke-on-Trent, GBR; 2 Radiology, Newcastle Teaching Hospitals, Newcastle upon Tyne, GBR; 3 Neuroradiology, Newcastle Teaching Hospitals, Newcastle upon Tyne, GBR; 4 Department of Molecular Genetics, Erasmus University Medical Center, Rotterdam, NLD

**Keywords:** predictive model, clinical outcome, mortality, endovascular therapy, tandem occlusion

## Abstract

Background: Tandem occlusions are intracranial large vessel occlusions (LVOs) with a concomitant ipsilateral extracranial internal carotid artery occlusion and can cause more severe stroke symptoms.

Aim: To develop a simple, rigorously cross-validated novel tool to predict clinical outcomes following tandem occlusion in patients with acute LVO stroke, based on data that are easily available to clinicians. To have used machine learning approaches to utilize the available information from clinical and angiographic data to make predictive models able to distinguish between mortality versus survival and good (modified Rankin Scale (mRS) ≤ 2) versus unfavorable neurological outcomes (mRs ≥ 3)

Materials and methods: Retrospective data from 87 consecutive patients with anterior circulation stroke and tandem occlusions who underwent mechanical thrombectomy and stenting between December 2009 and January 2020 were analyzed. Patients were stratified into three groups based on the location of their LVO, and these groups were compared using statistical tests. Predictive models were built and cross-validated 1000 times to estimate their predictive power, measured by accuracy and area under the receiver operating curve (AUROC).

Results: For distinguishing good outcome (mRS ≤ 2) versus poor outcome (mRS ≥ 3), the model comprised age, initial National Institute of Health Stroke Scale (NIHSS) score, Alberta Stroke Program Early CT Score (ASPECTS), NIHSS at 24 hours, NIHSS at discharge and intracranial haemorrhage and yielded an accuracy of 83% and the AUROC of 0.91. For mortality prediction, the model comprised age, initial NIHSS, intravenous thrombolysis, NIHSS at 24 hours and NIHSS at discharge and yielded an accuracy of 91% and an AUROC of 0.94.

Conclusions: Models developed exhibit strong predictive performance and can distinguish between both the instances of survival versus mortality and good versus poor outcome with an aim to support clinicians in deciding on optimal management for these complex patients. The developed model will help identify those at risk of poorer outcomes and the prospective better selection of patients with acute ischaemic large vessel stroke secondary to tandem occlusions.

## Introduction

Tandem occlusions (TO) are intracranial large vessel occlusions with a concomitant ipsilateral extracranial internal carotid artery (eICA) occlusion or high-grade stenosis [1-4]. They represent up to 30% of acute ischaemic strokes secondary to large vessel occlusion (LVO) [5-9] and can cause more severe stroke symptoms than either isolated intracranial or extracranial disease alone, with a poorer prognosis [3,6,9,10]. Rates of severe disability or death are reported in up to 69% of patients without treatment, with risks of developing large infarctions and morbidity and mortality rates of 70% and 55%, respectively [5,6]. Patients had a poor response to intravenous thrombolysis (IVT) alone with subsequent poor outcomes [7,8]. Reported rates of recanalization are 9%, favourable outcomes 30% and mortality rates are 22% to 50% [6,8].

Despite mechanical thrombectomy (MT) becoming the standard of treatment for acute ischaemic stroke in LVO, following the reported efficacy in recent large, randomised control trials and the prevalence of TO, tandem occlusions are either underrepresented or excluded from these studies [7,8,11] (32.3% in MR CLEAN, 18.6% in REVASCAT, 17% in ESCAPE and excluded from SWIFT-PRIME and EXTEND-IA [6]). Therefore, the evidence is lacking to optimise and standardise treatment [6]. Subgroup analyses of these trials have shown good results with MT in tandem occlusions [12], with some authors reporting equivalent outcomes of MT in LVO with or without an extracranial tandem occlusion and good recanalization rates following endovascular treatment [4,6]. However, multiple treatment options are discussed in the literature, with no clear consensus as to the optimal treatment option, particularly for the eICA disease [2,5,8].

To the best of our knowledge there are only two studies currently in print regarding identification of important predictors of good outcome as defined by modified Rankin Scale (mRS) score of 2 or less post tandem occlusion [13,14]. However, neither of these studies builds and validates the predictive model based on the statistically significant associations reported and both focus on good outcome alone while providing no information on mortality.

We conducted a retrospective observational study of patients from a single neurointerventional centre presenting with acute anterior circulation ischaemic stroke secondary to LVO and simultaneous ipsilateral eICA occlusion or high-grade stenosis that underwent endovascular treatment. We have used machine learning approaches to utilize the available information from clinical and angiographic data points to build predictive models able to distinguish between mortality versus survival and good (mRS ≤ 2) versus unfavourable neurological outcomes (mRS ≥ 3), thus adding to the growing body of scientific evidence base aiming to support clinicians in deciding on optimal management for these complex patients.

## Materials and methods

Between December 2009 and January 2020, consecutive patients treated in a single neurointerventional centre for acute ischaemic stroke and undergoing MT were identified retrospectively from the Thrombectomy register and data collected. The target population included patients with acute large vessel anterior circulation stroke and presenting with TO. The inclusion criteria for this study were patients that were treated for acute anterior circulation ischaemic stroke, having ipsilateral intracranial and extracranial occlusions or stenosis and undergoing endovascular treatment with MT for intracranial occlusion and with stenting of the extracranial disease. Patients with posterior circulation strokes, patients with anterior circulation strokes without TO and patients where MT was not performed were excluded from the study.

Data was collected for the patients for the following demographic and clinical data points: age, sex, smoking status, dyslipidemia, hypertension, diabetes mellitus and prior antiplatelet treatment. Admission scores for National Institute of Health Stroke Scale (NIHSS) and Alberta Stroke Program Early CT Score (ASPECTS) were recorded. Collected procedural data included use of bridging intravenous thrombolysis, using an antegrade or retrograde internal carotid artery (ICA) stenting approach, which consisted of treating the extracranial lesion first before the intracranial lesion, or vice versa; moreover, if the MT was performed using a stent retriever (SR), direct aspiration catheter (DAC) or combination of the two. Number of passes for the MT recorded. Inclusion in the study did not affect or influence the neurointerventional approach or management and patient inclusion was purely observational. The authors declare that Ethics Committee approval is not required, because this is a retrospective observational study based on anonymous, non-identifiable data for which the patients gave an informed verbal consent, and which is available from the corresponding author upon reasonable request.

Outcomes recorded were angiographic, functional and safety. Angiographic outcomes included end procedural revascularization, as measured by the modified Thrombolysis in Cerebral Infarction (mTICI) with a score of 2B-3 representing successful revascularization and a score of 3 as complete revascularization. Safety outcomes included embolisation into a new vascular territory (ENT), iatrogenic dissection and haemorrhage. Intracranial haemorrhage (ICH) was split into asymptomatic and symptomatic (sICH) which comprised any new intracranial haemorrhage and a deterioration in NIHSS score of four or more points [1,2,5]. Ninety-day mortality and functional outcomes were recorded, with a good neurological outcome being a mRS score of 2 or less.

Patients were stratified into three groups according to the location of their acute large vessel occlusion. These were either an intracranial occlusion of M1 or M2 segments of the middle cerebral artery (MCA) and critical stenosis of the eICA (NASCET [15]), tandem occlusions of the intracranial M1 or M2 MCA and complete cervical ICA occlusion or a single ICA occlusion extending intracranially, including carotid T-occlusions. The three groups were compared with respect to procedures and outcomes using χ2 test or one-way ANOVA to test for statistically significant differences, as appropriate. Predictive models were subsequently built and cross-validated 1000 times to get robust estimates of predictive power as measured by accuracy and Area Under the Receiver Operating Curve (AUROC). Statistical significance was accepted at 0.05 levels.

Development of statistical models

We shall develop predictive models based on multivariate logistic regression able to accurately distinguish between instances of survival versus mortality and good outcome (mRS ≤ 2) versus poor outcome (mRS ≥ 3). In both cases the models shall comprise only of variables found to be statistically significantly associated with corresponding outcome in univariate logistic regressions. We note that statistical significance will be accepted at the 0.05 level. This modelling approach ensures that predictive models are based only on the information of highest statistical importance which further enhances robustness and generalizability of the findings. All models will be built using the R statistical software tool (R Foundation for Statistical Computing, Vienna, Austria) [16]. Predictive performance of the models will be tested by running 1000 cross-validations on the dataset. Models will be learned on the training set and subsequently tested on the test set, which will amount to roughly 8700 test points for both models. Each cross-validation will be performed by splitting the entire dataset into training (90%) and test sets (10%). We will report both average accuracy and average AUROC over the test sets induced by the cross-validation procedure specified above.

## Results

A total of 87 patients undergoing endovascular treatment for anterior circulation acute ischaemic stroke at our centre underwent acute carotid stenting (ACS). These patients were stratified into three groups according to the location of their acute large vessel occlusion.

Group A had 26 patients with an intracranial occlusion (M1 or M2 segments of the middle cerebral artery) and critical stenosis of the eICA. Group B had 38 patients with occlusion of the intracranial M1 or M2 segments and an ipsilateral total occlusion of the cervical ICA. Group C had 23 patients with an isolated ICA occlusion extending intracranially, including carotid T-occlusion. The NIHSS score, presenting ASPECTS score and age were similar among patients (Table 1), with no significant difference between the groups. Sixty-four (74%) patients overall received intravenous thrombolysis prior to endovascular treatment, again not differing between the groups.

**Table 1:** Categorical data is summarized via raw counts and percentages n (%). Continuous variables were tested for normality by means of Kolmogorov-Smirnov test and were summarized in terms of mean (SD) or median (IQR), as appropriate. We have used χ2 test and Fisher’s exact test (in case of low expected counts) for categorical data and one-way ANOVA for continuous data; statistical significance was accepted at p<0.05 level [16]. a: One-way ANOVA b: χ2 test c: Fisher’s exact test NIHSS: National Institutes of Health Stroke Scale ASPECTS: Alberta Stroke Program Early CT Score IVT: intravenous thrombolysis SR: stent retriever DAC: distal access catheter mTICI: modified Thrombolysis in Cerebral Infarction ENT: embolisation into a new territory ICH: intracranial haemorrhage mRS: modified Rankin Scale

Variable/Outcome	Overall	Group A	Group B	Group C	p-value
	(n=87)	(n=26)	(n=38)	(n=23)	
Patients					
Age, y; mean (SD)	64.6 (10.7)	63.9 (12.2)	65.3 (10.8)	64 (8.8)	0.838^a^
Men	61 (70.1)	21 (80.8)	22 (57.9)	18 (78.2)	0.089^b^
Hypertension	43 (49.4)	11 (42.3)	22 (57.9)	10 (43.5)	0.379^b^
Diabetes mellitus	16 (18.4)	5 (19.2)	7 (18.4)	4 (15.4)	0.987^c^
Dyslipidaemia	26 (29.9)	8 (30.8)	12 (31.6)	6 (26.1)	0.896^b^
Current smoker	27 (31.0)	8 (30.8)	11 (28.9)	8 (34.8)	0.892^b^
Antithrombotics	62 (71.3)	21 (80.8)	25 (65.8)	16 (69.6)	0.420^b^
Admission NIHSS; mean (SD)	17.7 (6.5)	16.8(7.3)	18.0 (6.4)	17.9 (5.9)	0.730^a^
ASPECTS <7	24 (27.6)	9 (34.6)	8 (21.1)	7 (30.4)	0.461^b^
ASPECTS; median (IQR) or mean (SD)	7(6-9)	7.4(2.8)	7.5(1.8)	6.9 (3.0)	0.630^a^
Approach					
Antegrade	14 (17.8)	6 (23.1)	5 (13.2)	3 (13.0)	0.512^c^
Retrograde	65 (82.2)	18 (69.2)	33 (86.8)	14 (60.9)	0.058^b^
IVT	64 (73.6)	19 (73.1)	24 (63.2)	21 (80.8)	0.054^b^
Device					
SR	48 (55.2)	20 (76.9)	20 (52.6)	8 (34.8)	0.013^a^
DAC	23 (26.4)	3 (11.5)	11 (28.9)	9 (39.1)	0.082^b^
Combined	12 (13.8)	3 (11.5)	7 (18.4)	2 (8.7)	0.523^a^
Angiography					
mTICI 2B/3	79 (90.8)	25 (96.2)	33 (86.8)	21 (91.3)	0.447^a^
mTICI 3	35 (40.2)	13 (50.0)	14 (36.8)	8 (34.8)	0.473^b^
First Pass	41 (47.1)	12 (46.2)	19 (50.0)	10 (43.5)	0.879^b^
Complications					
ENT	4 (4.6)	2 (7.7)	2 (5.3)	0 (0.0)	0.424^c^
Iatrogenic dissection	7 (8.0)	2 (7.7)	4 (10.5)	1 (4.3)	0.688^c^
Total ICH	15 (17.2)	7 (26.9)	3 (7.9)	5 (21.7)	0.113^c^
Symptomatic ICH	5 (5.7)	2 (7.7)	2 (5.3)	1 (4.3)	0.869^c^
NIHSS at discharge; median(IQR) or mean (SD)	10.5 (2-20.8)	10.8 (10.5)	14.8 (13.7)	14.8 (13.7)	0.330^a^
90-day outcomes	(n=85)	(n=24)	(n=38)	(n=23)	
mRS ≤2	39 (45.9)	14 (58.3)	16 (42.1)	9 (39.1)	0.343^b^
Died	18 (21.2)	3 (12.5)	11 (28.9)	4 (17.3)	0.265^c^

The majority of patients (65; 82%) were treated with a retrograde approach with mechanical thrombectomy of the intracranial occlusion performed in the first instance followed by stenting of the extracranial ICA. The method of MT was at the discretion of the operating neurointerventionalist, with a tendency towards the use of stent retrievers only in patients in Group A, whilst there was a more varied approach to MT in Group B patients. In Group C four patients did not require MT once the carotid had been stented in an antegrade fashion.

Angiographic outcomes were similar between the groups. In Group A, 25 out of 26 patients (96%) achieved successful reperfusion with a mTICI score of 2B/3 with a median time of symptom onset to revascularization of 300 minutes; 12 (46%) were first-pass recanalization. In Group B, 33 out of 38 patients (87%) achieved successful reperfusion of mTICI 2B/3 with a median time of 310 minutes from symptom onset to revascularization and 19 (50%) were successful on first pass. Finally in Group C, 21 out of 23 patients (91%) achieved successful reperfusion with a mTICI 2B/3 with a median time of 326.5 minutes from symptom onset to revascularization and 10 (44%) on first-pass thrombectomy.

ENT occurred in two patients each in Group A and Group B, with no specific MT strategy seen as associated (two were using an SR + DAC combination, one using SR only and one using DAC only). Stent failure occurred in one case, where a Cristallo stent did not adequately deploy but this was rescued with angioplasty and the patient went on to achieve a good neurological outcome at 90 days. Iatrogenic dissection was observed in seven cases and was managed with stenting in each case.

There were two symptomatic ICH in each of Group A and Group B, with a further symptomatic ICH in Group C. These represented a symptomatic ICH rate of two (7.7%), two (5.3%) and one (4.3%) for each of the groups respectively, with no significant difference between the groups. Interestingly, ICH was noted in five out of 14 antegrade patients (36%) although only one of these cases was symptomatic (7%). There were 10 (16%) ICH cases in the 64 patients treated with a retrograde approach of which four (6%) were symptomatic. The overall sICH rate was five (5.7%).

Ninety-day outcomes were available for 24 of the 26 Group A patients and all of the patients from Groups B and C, with overall just two patients being lost to follow-up.

Good neurological outcomes were more frequently seen in Group A, with 14 (58%) patients achieving an mRS ≤ 2 at 90-day follow-up; however, this was not significant when compared to the rates in both of the other groups. There was also a non-significant trend towards higher 90-day mortality in the Group B patients who had total ICA occlusion. Good neurological outcomes were comparable among the patients treated with antegrade and retrograde approaches (nine (52%) and 30 (43%) respectively).

Statistical model analysis

Using the retrospectively collected data from 87 cases, statistical analysis was performed to develop predictive models based on multivariate logistic regression able to accurately distinguish between instances of survival versus mortality and good outcome (mRS ≤ 2) versus poor outcome (mRS ≥ 3). In both cases the models comprise only variables found to be statistically significantly associated with the corresponding outcome found in univariate logistic regressions to enhance robustness of the findings. To be more specific, in the case of survival prediction, only the variables age, initial NIHSS, prior IVT, NIHSS at 24 hours and NIHSS at discharge were found to be significantly associated with mortality while the remaining variables did not exhibit significant association (summary presented in Table 2).

**Table 2:** Odds ratios, their respective confidence intervals (CI) and p values for the variables comprising the model for mortality prediction. NIHSS: National Institute of Health Stroke Scale

Variable	Odds Ratio	95% CI for Odds Ratio	p value
Age	1.07	(1.02,1.14)	0.02
Initial NIHSS	1.16	(1.05,1.30)	0.006
Thrombolysis	0.25	(0.08,0.76)	0.01
NIHSS at 24 hours	1.18	(1.10,1.30)	3.91×10^-5^
NIHSS at discharge	1.22	(1.14,1.37)	7.33×10^-6^

**Figure 1:**
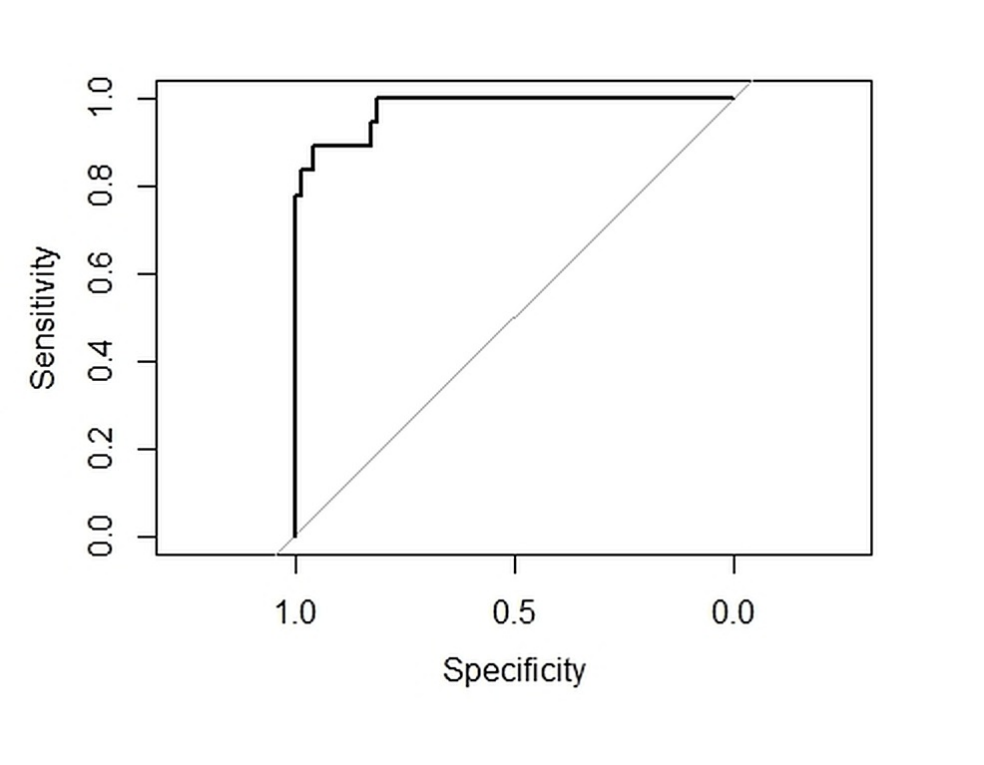
Receiver operating curve showing the sensitivity and specificity of our survival prediction model based on the variables summarised in Table 2.

This model, upon 1000 cross-validations, exhibited an average accuracy of 91% and an average AUROC of 0.94 (Figure 1).

Additionally, in the case of distinguishing good neurological outcome (mRS ≤ 2) versus poor outcome (mRS ≥ 3), only the variables age, initial NIHSS, ASPECTS score, NIHSS at 24 hours, NIHSS at discharge and haemorrhage were found to be significantly associated with the mRS while the remaining variables did not exhibit significant association (summary presented in Table 3).

**Table 3:** Odds ratios, their respective confidence intervals (CI) and p values for the variables comprising the model aimed at distinguishing between good neurological outcome (mRS ≤ 2) and poor neurological outcome (mRS ≥ 3). mRS: modified Rankin Score NIHSS: National Institute of Health Stroke Scale ASPECTS: Alberta Stroke Program Early CT Score

Variable	Odds Ratio	95% CI for Odds Ratio	p value
Age	0.94	(0.90,0.98)	0.006
Initial NIHSS	0.90	(0.84,0.97)	0.007
ASPECTS	1.36	(1.07,1.78)	0.02
NIHSS at 24 hours	0.77	(0.69,0.85)	1.66×10^-6^
NIHSS at discharge	0.80	(0.71,0.87)	5.47×10^-6^
Haemorrhage	0.66	(0.42,0.97)	0.05

**Figure 2:**
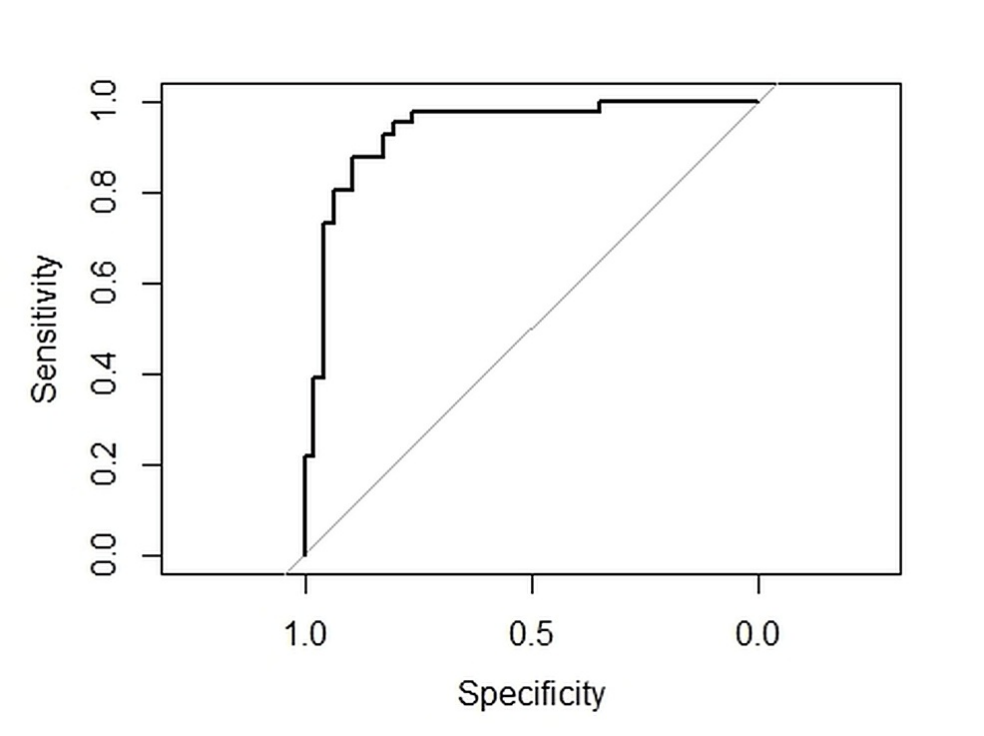
Receiver operating curve showing the sensitivity and specificity of good neurological outcome (mRS ≤ 2) versus poor outcome (mRS ≥ 3) based on variables summarized in Table 3. mRS: modified Rankin Score

This model exhibited, upon 100 cross-validations, an average accuracy of 83% and an average AUROC of 0.91 (Figure 2). A detailed description of the cross-validation procedure is outlined in the methodology section “Development of statistical models”. 

## Discussion

Several large prospective randomised controlled trials have proven the safety and efficacy of mechanical thrombectomy in acute ischaemic stroke [17] with improved recanalization rates and functional outcomes [5,8]. It is now the standard treatment [8].

Up to 30% of patients with acute ischaemic stroke secondary to intracranial large vessel occlusion have additional, ipsilateral disease of the cervical carotid artery [15], either occlusion [1-3,6,18] or high-grade stenosis [4,8,19]. These tandem occlusions do poorly with IVT alone [5,19], yet, due to their complex nature [5], they are often excluded from [2,5] or underrepresented in the large trials [6], therefore highest level of scientific evidence to support treatment options is lacking [3,19,20]. Sub-analysis of the large trials found that TO did better with MT [19], with good outcomes reported after endovascular treatment [2,5,21]. HERMES, for example, demonstrated similar outcomes for TO patients after MT to those with isolated LVO [2]. TO treatment, however, is more complex than isolated LVO [5] as TO treatment requires tackling the extracranial occlusion. The procedure typically consists of combining mechanical thrombectomy with extracranial recanalization in the form of stenting or angioplasty [17]. 

The optimal method is much disputed in the literature, with no comparative studies to establish superiority of either approach [20]. HERMES, for example, did not report on the optimal treatment strategy for extracranial disease [2]. Randomised controlled trials are needed for this purpose [2].

Outcomes frequently observed by studies included angiographic (successful reperfusion [1,2,8]) functional (good neurological outcomes [1,5,8,22]) and safety (symptomatic intracranial haemorrhage [1,2,8,12,22], death [1,2,5,8,12,23] and procedural complications [2,23]).

For our patient cohort the overall rates of mTICI 2B/3 were 79 (90.8%) and mTICI 3 were 35 (40.2%), with no significant difference between the groups. Again, these were in line with those reported in the literature (Bricout et al. 61.5% 2B/3 [12], Heck and Brown 2B/3 74% [20], Behme et al. 2B 77% [7], Fernández Menéndez et al. 87.8% [6]).

We observed a rate of ENT of four (4.6%). This is consistent with rates reported in Blanc et al. (6%) [23] and in the large, randomised trials (EXTEND-IA 5.7%; ESCAPE 5%; REVASCAT 4.9%; MR CLEAN 8.6%, of which 5.6% had ischaemic stroke symptoms in a new vascular territory [22]). Despite these rates, these large trials did not comment on the outcomes of those affected (except ESCAPE who had worse clinical outcomes) [24].

We report an overall mortality rate of 18 (21.2%), again with no significant difference between either our groups or from figures cited in the literature (Fernández Menéndez et al. 20.2% [6], Kappelhof et al. 45% [1], Behme et al. 19% [7], Blanc et al. 23% [23]). Fernández Menéndez et al. [6] found association between certain variables and survival, with coronary heart disease, longer times to recanalization, no recanalization and higher admission NIHSS scores were independently associated with mortality [6]. In the TITAN cohort, Zhu et al. [2] identified predictors of 90-day mortality as age, current smoking, NIHSS and ASPECTS scores and prior IVT [2]. Hellegering et al. [8] found that age, treatment duration and recanalization times were independently associated with all-cause mortality, with prior IVT and stroke severity also associated [8]. In line with these findings, we found that age, NIHSS scores and IVT were among the variables univariately significantly associated with the instances of mortality and were subsequently included in the corresponding predictive model.

We have identified only two studies [13,14] already in print that focus on identifying independent predictors of good outcomes in similar patient cohorts. To be more specific it is reported that age, NIHSS, ASPECTS, mTICI 3 and hypertension are univariately statistically significantly associated with the instances of good outcome post-endovascular treatment of tandem occlusion [14]. Equally, the authors find that age, admission NIHSS, hypertension, atrial fibrillation, prior IVT and sICH are univariately statistically significantly associated with the instance of good outcome post-endovascular treatment of patients with tandem lesion in the anterior circulation [13]. In line with these findings, we found that age, NIHSS score, and ASPECTS score were among the variables univariately significantly associated with the instances of good outcome and were subsequently included in the corresponding predictive model.

However, none of the studies mentioned [2,6,8,13,14] make an attempt to build and validate the predictive model based on the significant variables identified. To the best of our knowledge, this is the first study that builds and rigorously cross-validates predictive models for the identification of instances of survival and good outcomes upon endovascular treatment of tandem occlusions.

Model selection was performed to develop predictive models based on multivariate logistic regression based on univariately statistically significant variables alone for both survival and distinguishing between good outcome (mRS ≤ 2) and poor outcome (mRS ≥ 3). In the case of mortality prediction, the model comprised of age, initial NIHSS, thrombolysis, NIHSS at 24 hours and NIHSS at discharge yielded an accuracy of 91% and an AUROC of 0.94.

Moreover, in the case of distinguishing good outcome (mRS ≤ 2) versus poor outcome (mRS ≥ 3), the model comprised of age, initial NIHSS, ASPECTS, NIHSS at 24 hours and NIHSS at discharge and intracranial haemorrhage yielded an accuracy of 83% and an AUROC of 0.91.

There is a significant body of knowledge already in print on the research topic of tandem occlusions and treatment options; however, few examples of statistical models were used in this setting to predict outcomes post-treatment. Modelling tools, such as prognostic models presented in this manuscript, can predict certain outcomes and aid in the decision-making process [25], helping to develop patient-centred care, tailor management and identify those at greater risk for poorer outcomes or complications needing specific management. Whilst these prognostic models were based on a limited number of patients, they equally comprise variables found to be statistically significantly associated with favourable outcomes post-endovascular treatment of tandem occlusion in larger patient cohorts [2,6,8,13,14] which enhances our confidence in the external applicability of the models created. Finally, these models show promise for providing accurate prediction of patient outcomes based on easily collectible clinical data points, helping to guide management and identify patients most at risk.

Limitations

Limitations of our study include a single-centre patient cohort of retrospectively analysed patients. Our selection criteria limit our analysis of the comparative efficacy of different treatments as it included TO patients undergoing emergency carotid stenting only. Whilst this adds additional evidence to support efficacy and safety of stenting in TO, large randomised controlled trials are needed to truly establish the optimum treatment strategy.

## Conclusions

Despite the revolution of mechanical thrombectomy, tandem occlusions present a unique challenge as they pose a significant barrier to maximising revascularization and thus affect the clinical outcomes of patients with acute ischaemic stroke. The clinical outcomes for patients with tandem occlusions can be varying and there is no definitive predictive model that can help with better patient selection. The models developed in our study can be used as a part of this solution. The developed models help by maximising patient selection as they exhibit strong predictive performance and are able to distinguish between both the instances of survival versus mortality and good versus poor outcome with an aim to support clinicians in deciding on optimal management for these complex patients.

We have laid the initial foundation for this complex condition by developing a model that will help identify those at risk of poorer outcomes and the prospective better selection of patients with acute ischaemic large vessel stroke secondary to tandem occlusions.
